# Krill Oil in Human Nutrition and Healthy Aging: A Narrative Review of Implications for Intrinsic Capacity and Nutrient Bioavailability

**DOI:** 10.3390/nu18162622

**Published:** 2026-08-11

**Authors:** Andrea Rodrigues Vasconcelos, Denise Deo Dias, Ana Carolina Remondi Souza, Erlon Oliveira de Abreu-Silva, Aline Marcadenti, José João Name

**Affiliations:** 1 Kilyos Assessoria, Cursos e Palestras, São Paulo 01311-100, Brazil; andrea.vasconcelos@kilyos.com.br (A.R.V.); denise.dias@kilyos.com.br (D.D.D.); anacarolina.souza@kilyos.com.br (A.C.R.S.); 2 Hcor Research Institute (IP-Hcor), Coração Associação Beneficente Síria, São Paulo 04005-909, Brazil; erlon@terra.com.br (E.O.d.A.-S.); marcadenti@yahoo.com.br (A.M.); 3 Division of Health Care Sciences, Dresden International University, 01307 Dresden, Germany; 4 Graduate Program in Health Sciences (Cardiology), Instituto de Cardiologia/Fundação Universitária de Cardiologia do Rio Grande do Sul (IC/FUC), Porto Alegre 90620-001, Brazil; 5 Undergraduate Nutrition Program, Faculdade de Ciências Médicas da Santa Casa de São Paulo, São Paulo 01225-010, Brazil

**Keywords:** krill oil, marine phospholipids, bioavailability, healthy aging, intrinsic capacity

## Abstract

Krill oil (KO) is a marine-derived nutritional ingredient distinguished by its unique structural composition and increasing relevance in human nutrition, particularly in the context of healthy aging and preservation of intrinsic capacity. Approximately 30–65% of its fatty acids are esterified to phospholipids, a molecular configuration associated with enhanced bioavailability and efficient tissue incorporation of omega-3 polyunsaturated fatty acids, even at relatively low doses. Antarctic krill (*Euphausia superba*) originates from minimally impacted marine ecosystems, contributing to a favorable safety profile characterized by inherently low levels of environmental contaminants, including heavy metals and persistent organic pollutants. Beyond its structural and safety attributes, KO provides a complex nutrient matrix comprising approximately 25% total *n*-3 polyunsaturated fatty acids (PUFAs), including ~14% eicosapentaenoic acid (EPA) and ~6.5% docosahexaenoic acid (DHA), together with choline and astaxanthin. This compositional synergy may modulate inflammatory pathways, redox balance, membrane dynamics, and cellular signaling, thereby influencing physiological systems implicated in aging biology. This narrative review critically examines how these distinctive characteristics translate into clinically relevant outcomes supporting the multifunctional applications of KO in human nutrition. The analysis is structured within the framework of intrinsic capacity, encompassing locomotion, vitality, sensory, cognition and psychological dimensions, as a conceptual model for healthy aging. Considerations on bioavailability, tolerability, and sustainability are also discussed in the context of nutritional strategies promoting healthy longevity.

## 1. Introduction

Aging is a complex and multifactorial process characterized by progressive physiological decline across multiple systems, including cardiovascular, metabolic, musculoskeletal, and neurological functions. These changes contribute to reduced functional reserve and increased vulnerability to stressors, with marked interindividual variability that underlies differences in resilience and susceptibility to frailty [1].

The global population is aging at an unprecedented pace and is living longer, reshaping demographic structures and increasing pressure on health systems worldwide. According to the World Health Organization (WHO), the number of individuals aged 60 years and older is projected to rise from 1 billion in 2020 to 2.1 billion by 2050 [2]. This demographic shift reinforces the need for strategies that extend not only longevity but also functional capacity and quality of life in later years. In this context, the WHO has proposed the concept of intrinsic capacity (IC) as the composite of an individual’s physical and mental capacities that underpin healthy aging. Because IC reflects the integrated functioning of multiple physiological systems, its preservation is strongly influenced by modifiable lifestyle-related factors, particularly nutrition. Accordingly, bioactive nutrients with anti-inflammatory and antioxidant properties may play a key role in mitigating biological processes associated with age-related functional decline.

Several biological processes associated with age-related functional decline, including chronic low-grade inflammation, oxidative imbalance, mitochondrial dysfunction, and impaired vascular and neuromuscular function, are potentially modifiable through nutritional interventions. Accordingly, marine-derived bioactive lipids have emerged as biologically plausible candidates for modulating pathways implicated in aging-related physiological deterioration. Among these, omega-3 polyunsaturated fatty acids (*n*-3 PUFAs), particularly eicosapentaenoic acid (EPA) and docosahexaenoic acid (DHA), have been extensively investigated due to their role in inflammation regulation, membrane fluidity, neuronal signaling, and cellular homeostasis [3]. The biological relevance of long-chain *n*-3 PUFAs is further supported by their preferential accumulation in tissues highly susceptible to aging-related dysfunction, including the brain, retina, and cardiovascular system [4]. However, in Western populations, circulating levels of EPA and DHA are frequently reported below proposed optimal ranges for cardiometabolic and neurological health [5,6]. This pattern is largely attributable to insufficient dietary intake combined with the limited endogenous conversion of α-linolenic acid (ALA) into its long-chain derivatives. Under typical physiological conditions, ALA conversion to EPA is relatively low, whereas conversion to DHA is virtually negligible [5,7]. Consequently, achieving adequate physiological levels of EPA and DHA relies primarily on dietary sources, driving increasing interest in alternative and sustainable marine-derived lipid sources.

Among emerging marine-derived lipid sources, krill oil (KO) has attracted growing interest due to its distinctive lipid composition and complex bioactive matrix. KO provides *n*-3 PUFAs, including EPA and DHA [5,8,9], together with naturally occurring astaxanthin, choline, vitamins, minerals, and flavonoids, which may collectively influence their bioavailability and metabolic effects [10]. These compositional characteristics have positioned KO as a potentially relevant nutritional strategy for modulating biological processes associated with aging-related functional decline.

However, despite increasing interest in KO supplementation, its potential relevance within the framework of IC and healthy aging has not been comprehensively discussed. Therefore, this narrative review synthesizes and critically discusses current evidence regarding the structural, biochemical, and functional properties of KO, with emphasis on how its unique nutrient matrix may modulate biological pathways associated with the preservation of physiological function during aging.

## 2. Methods

This narrative review was conducted in accordance with current methodological recommendations and reporting principles for high-quality narrative reviews [11,12]. A structured literature search was performed using PubMed, Google Scholar, and other relevant scientific databases to identify relevant studies evaluating the biochemical characteristics, bioavailability, safety, and physiological effects of KO. Additional articles were identified through manual screening of reference lists from relevant reviews and original studies.

Search terms combined terms related to KO (“krill oil”, “Antarctic krill oil”, “omega-3 phospholipids”) with terms related to aging and intrinsic capacity domains, including locomotion, vitality, cognition, sensory function, and psychological well-being, as well as related outcomes. The review focused primarily on randomized controlled trials, human intervention studies, and clinically relevant evidence published in peer-reviewed journals. For clinical evidence, studies involving pediatric populations, non-randomized interventions, outcomes unrelated to intrinsic capacity domains, or interventions combining KO with other bioactive compounds were excluded to improve the interpretability of KO-specific effects. No restrictions were applied regarding year of publication or language. The literature search was conducted between February and July 2026.

Although a structured literature search and predefined study selection criteria were applied to identify and select relevant studies, this review was not conducted or registered as a formal systematic review, and no meta-analysis was performed. Rather than generating pooled quantitative estimates, the primary objective was to provide a critical narrative synthesis and contextual interpretation of the available evidence regarding KO within the framework of intrinsic capacity and geroscience-oriented nutritional strategies for healthy aging.

## 3. Krill Oil

The Antarctic krill (*Euphausia superba* Dana) is a pelagic crustacean endemic to the Southern Ocean and represents one of the largest biomasses among multicellular organisms [13,14,15,16,17,18,19]. Beyond its ecological importance within the Antarctic food web, krill has attracted increasing scientific and commercial interest as a marine source of *n*-3 PUFAs and other bioactive compounds [10,20,21]. The sustainability of Antarctic krill fisheries is regulated by the Commission for the Conservation of Antarctic Marine Living Resources (CCAMLR), which establishes precautionary catch limits aimed at minimizing ecological impacts on Antarctic ecosystems [14,22,23,24]. Current harvesting levels remain substantially below the established catch thresholds, while Antarctic krill fisheries are generally associated with comparatively low bycatch rates relative to many conventional marine fisheries [17,23,25].

Krill-derived oil is characterized by a distinctive lipid composition rich in long-chain *n*-3 PUFAs, notably EPA (20:5*n*-3) and DHA (22:6*n*-3), which originate from marine microalgae and accumulate through the Antarctic food chain [10]. Unlike conventional fish oil (FO), in which EPA and DHA are predominantly esterified in triglycerides, KO contains approximately 25% total *n*-3 polyunsaturated fatty acids (PUFAs), including ~14% eicosapentaenoic acid (EPA) and ~6.5% docosahexaenoic acid (DHA), of which approximately 30–65% are esterified to phospholipids, especially phosphatidylcholine [4,8,9,17,18,25,26]. A comparative overview of the major bioactive constituents of KO reported in representative studies is presented in Table 1, highlighting the compositional variability associated with different extraction methods and processing conditions. Due to the high activity of endogenous proteolytic enzymes, krill biomass requires rapid post-harvest processing to minimize autolysis and preserve oil quality [10].

In addition, KO naturally contains astaxanthin, a xanthophyll lipophilic carotenoid responsible for its characteristic reddish coloration [4]. Astaxanthin exhibits potent antioxidant and anti-inflammatory properties that may contribute both to the oxidative stability of the oil and to its biological activity [30,31]. Mechanistically, its molecular structure enables efficient scavenging of reactive oxygen and nitrogen species (RONs) and interactions with cellular membrane systems [32].

Beyond *n*-3 PUFAs and astaxanthin, KO also provides choline, primarily in the form of phosphatidylcholine, which is involved in membrane structure, lipid transport, and one-carbon metabolism [18]. These compositional characteristics further contribute to the nutritional and metabolic relevance of KO, complementing the well-established physiological effects of EPA and DHA, particularly their role in triglyceride lowering [33,34].

Taken together, the distinctive molecular composition of KO, combined with the presence of endogenous bioactive constituents such as astaxanthin and choline, supports its growing relevance as a marine-derived nutritional ingredient. Although primarily marketed as a dietary supplement, KO also has potential as a novel food ingredient; however, its application in food matrices remains underexplored, warranting further investigation into its stability, biological activity, and functionality [25,35]. For a broader overview of the utilization of Antarctic krill and krill-derived products in human nutrition, the relevant literature should be consulted [36,37,38,39]. Among these characteristics, the phospholipid-bound organization of EPA and DHA has attracted particular attention due to its potential influence on the digestion, absorption, and tissue distribution of long-chain *n*-3 PUFAs, as discussed in the following section.

### 3.1. Bioavailability

The distinctive bioavailability profile attributed to KO is thought to derive primarily from its phospholipid-rich organization, in which phospholipids function as structural carriers for long-chain *n*-3 PUFAs and associated bioactive compounds. Because biological membranes are phospholipid-based structures, this molecular arrangement has been proposed to influence the digestion, absorption, transport, and tissue distribution of krill-derived omega-3 fatty acids relative to triglyceride-based marine oils [4,8,40]. Mechanistically, phospholipid-bound EPA and DHA may be more efficiently incorporated into mixed micelles during digestion, facilitating enterocyte uptake and subsequent incorporation into circulating lipoprotein fractions [4,41,42].

Short-term pharmacokinetic and crossover studies generally support a distinct absorptive profile for krill-derived *n*-3 PUFAs [4,43]. Compared with conventional triglyceride or ethyl ester FO formulations, KO supplementation has been associated with greater or more rapid incorporation of EPA and DHA into plasma phospholipid fractions, as reflected by higher area-under-the-curve responses [5,41]. Additional pharmacokinetic studies further suggest that phospholipid/free fatty acid formulations derived from KO may provide sustained systemic availability and reduced dependence on concomitant dietary fat intake for efficient absorption [44,45]. Together, these findings support the concept that phospholipid-rich omega-3 formulations may influence the early metabolic handling and systemic distribution of long-chain *n*-3 PUFAs.

The apparent absorptive advantage of KO is also supported by intervention studies showing comparable or greater biological responses despite lower absolute EPA and DHA doses. In an open label, single-center, randomized, parallel-group trial involving 113 healthy subjects, KO supplementation (3.0 g/day, providing 543 mg EPA + DHA) induced increases in circulating EPA and DHA comparable to those observed with FO (1.8 g/day, providing 864 mg EPA + DHA), despite delivering approximately 37% less *n*-3 PUFAs [8]. This suggests the enhanced metabolic utilization of krill-derived omega-3.

In line with this, a randomized crossover study with 24 individuals (mean age, 28.2 years) compared the effects of KO, FO, and corn oil administered for 4 weeks using matched omega-3 doses. This study demonstrated that KO supplementation resulted in significantly greater increases in plasma and red blood cell EPA and DHA levels, as well as a higher Omega-3 Index, compared to FO [40], further supporting a more efficient incorporation into circulating and membrane lipid pools. Notably, the phospholipid content of KO appears to be a key determinant of this effect. In a randomized crossover study comparing KO formulations with different phospholipid concentrations, supplementation with a high-phospholipid KO resulted in significantly greater incorporation of *n*-3 PUFAs into erythrocytes compared to a low-phospholipid formulation, despite equivalent omega-3 dosing [46]. Similarly, a recent randomized clinical trial evaluated the effects of KO and FO in 72 healthy adults (53 women and 19 men) receiving an equivalent dose of 1.1 g/day of *n*-3 PUFAs for 12 weeks. Compared with FO, KO produced approximately 1.5-fold greater increases in circulating EPA and DHA concentrations despite providing the same amount of *n*-3 PUFAs [47]. These findings provide mechanistic support for the role of phospholipids in enhancing the bioavailability and membrane incorporation of krill-derived *n*-3 PUFAs.

Importantly, not all comparative studies have demonstrated the superiority of KO over alternative phospholipid-containing omega-3 formulations. In a randomized, double-blind, crossover study involving 24 healthy adults aged 18–55 years, participants were allocated to receive either a phospholipid-enhanced fish oil (PEFO) formulation providing 201 mg EPA and 136 mg DHA or KO providing 135 mg EPA and 71 mg DHA. Acute administration of KO resulted in plasma EPA and DHA exposure comparable to a phospholipid-enhanced FO formulation, as reflected by the geometric mean ratio (GMR) for the 24-h incremental area under the curve (iAUC_0–24_h) (PEFO = 0.83; 90% CI: 0.60–1.15; *p* = 0.83). However, differences in absorption kinetics were observed, with the PEFO showing a higher and earlier EPA peak response during the first 10 h after ingestion (EPA iAUC_0–10_h: 113 vs. 81.6 nmol/mL·h for KO; GMR: 1.35; 90% CI: 1.16–1.57; *p* < 0.01). These findings suggest that phospholipid content itself, rather than marine source alone, may be a key determinant of omega-3 absorption dynamics [48].

Consistent with this interpretation, when comparable EPA and DHA intakes are administered over several weeks of supplementation durations, differences between lipid matrices appear to be attenuated. Randomized trials ranging from 4 to 12 weeks have reported similar plasma and erythrocyte omega-3 enrichment following supplementation with KO, FO, or other marine-derived lipid matrices when equivalent omega-3 doses were provided [49,50]. In line with these findings is a recent meta-analysis of 26 randomized controlled trials, conducted predominantly in healthy individuals but also including participants with overweight or obesity, dyslipidemia, type 1 diabetes, and older adults. Daily EPA + DHA intake was categorized as low-dose (100–1900 mg/day), medium-dose (2000–2900 mg/day), or high-dose (>3000 mg/day). The analysis suggested greater efficiency in omega-3 incorporation with KO at lower dosages (<2000 mg/day), whereas differences between formulations became less pronounced at higher or equivalent omega-3 intakes [51]. Collectively, these findings indicate that while KO may confer advantages in terms of bioavailability and dose efficiency, particularly at lower intakes, overall omega-3 enrichment appears to depend on multiple factors, including lipid structure, dosage, formulation, and duration of exposure.

Emerging lipidomic evidence further suggests that KO may influence the qualitative distribution of omega-3 fatty acids across specific lipid classes. Acute and long-term crossover studies conducted in healthy women demonstrated that KO consumption increased postprandial plasma levels of EPA and DHA predominantly within phospholipid fractions, as assessed by plasma lipidomic analyses. In contrast, omega-3 fatty acids derived from FO were more commonly incorporated into triglyceride-rich lipid fractions [52,53]. These findings suggest that the molecular form of omega-3 delivery may influence lipid remodeling and compartmentalization beyond conventional measures of systemic absorption.

Taken together, current evidence supports the notion that the phospholipid-rich matrix of KO may influence absorption kinetics, metabolic handling, and tissue distribution of long-chain *n*-3 PUFAs. Although the magnitude and clinical relevance of these differences remain incompletely resolved, available data support the concept that lipid structure contributes to the incorporation of *n*-3 PUFA into membrane-relevant lipid pools and may influence biological responses under specific nutritional and dosing conditions. The principal compositional differences and findings from representative human studies directly comparing KO and FO are summarized in Table 2.

### 3.2. Safety and Tolerability

KO is generally regarded as safe, as supported by toxicological evaluations, clinical studies, and regulatory approvals from major health authorities worldwide [10,17,35,57,58].

Preclinical studies consistently support a favorable toxicological profile for KO, characterized by low toxicity and absence of significant cytotoxicity effects at physiologically relevant concentrations [59]. Consistent with these findings, human clinical trials have reported good tolerability, with safety outcomes comparable to placebo across a range of supplementation protocols [17,33,60,61,62,63,64]. In large randomized clinical trials, KO supplementation has not been associated with serious adverse events, treatment discontinuation, bleeding events, serious gastrointestinal disorders, or significant changes in vital signs, electrocardiographic parameters, liver enzymes, or creatine kinase [33]. The most common reported adverse effects are mild gastrointestinal symptoms, including flatulence, heartburn, and diarrhea [17,60,65].

The phospholipid-rich structure of KO may also contribute to its gastrointestinal tolerability. Due to their amphiphilic properties, phospholipids facilitate dispersion in aqueous environments and may influence digestive behavior and lipid emulsification dynamics [66]. These physicochemical properties may enhance gastrointestinal dispersion and also contribute to minimizing the characteristic fishy aftertaste commonly associated with conventional FO supplements [4,18,67,68].

Although highly purified KO products are generally non-allergenic, caution is warranted in individuals with seafood allergies [69]. In addition, because KO provides long-chain *n*-3 PUFAs associated with reduced platelet aggregation and potential antithrombotic effects, caution may also be appropriate in individuals using anticoagulant or antiplatelet medications, although clinically significant bleeding events have not been consistently reported in interventional studies [17,70].

KO is also characterized by a comparatively low contaminant burden relative to many predatory marine fish species. Because Antarctic krill occupies a low trophic level and has a relatively short lifespan, the accumulation of environmental contaminants such as mercury and persistent organic pollutants (POPs) tends to be lower than that observed in higher-trophic-level marine organisms [17,18,71,72,73,74,75,76,77,78,79,80,81,82]. Although Antarctic ecosystems are not entirely free from anthropogenic pollutants, available evidence indicates that contaminant concentrations in krill-derived products generally remain low and within established safety limits.

Available analyses of finished krill-derived supplements provide quantitative context for their contaminant profile relative to recognized safety benchmarks. In three commercial krill supplements, total mercury concentrations of 7.3–17 μg/kg, cadmium concentrations of 0.42–0.82 mg/kg, and lead concentrations of 0.27–0.40 mg/kg corresponded to approximately 7.3–17%, 42–82%, and 9–13%, respectively, of the current European Union maximum levels for food supplements of 0.10, 1.0, and 3.0 mg/kg [83,84]. In a larger survey of 19 commercial KO products, the median estimated daily intakes of inorganic arsenic, cadmium, and lead were 0.073, 0.010, and 0.037 μg/day, respectively, corresponding to approximately 0.73%, 0.17%, and 0.19% of the NSF/ANSI Standard 173 (Dietary Supplements) limits applied in that study—10, 6, and 20 μg/day, respectively [85]. Regarding POPs, two commercial KOs consumed at the maximum recommended dose provided hexachlorobenzene (HCB), polychlorinated biphenyls (PCBs), and combined dioxin/furan/dioxin-like PCB toxic equivalents corresponding to 0.046–0.10%, 0.015–0.034%, and 0.18–0.23% of the respective tolerable daily intakes, while total dichlorodiphenyltrichloroethane-related compounds (ΣDDT) was detectable in only one product, at 0.0015% of the corresponding tolerable daily intake [65]. Overall, these findings indicate that the evaluated products remained within the applicable regulatory or health-based reference values; however, the limited number of market surveys and potential product-to-product variability preclude extrapolation to all commercial KOs and reinforce the importance of selecting well-characterized, quality-controlled products supported by appropriate contaminant testing.

## 4. Krill Oil Across Domains of Intrinsic Capacity

### 4.1. Conceptual Framework and Domains of Intrinsic Capacity

Most older adults remain in community settings, where they experience multidimensional health challenges associated with advancing age [86]. Age-related declines in muscle mass, strength, balance, sensory function, cognition, and psychosocial well-being progressively compromise mobility, nutrition status, autonomy, and social participation [86,87,88,89]. Together, these alterations affect interconnected functional domains, including locomotion, vitality, sensation, cognition, and psychological health, thereby undermining well-being and the ability to live independently in older age [86].

Historically, healthy aging was predominantly conceptualized as the absence of major chronic diseases. However, growing recognition of functional decline and heterogeneity in aging trajectories prompted a shift toward function-centered models of aging. In its World Report on Ageing and Health, the WHO redefined healthy aging as the process of developing and maintaining functional ability, thereby moving beyond disease-centered paradigms toward a framework focused on physiological capacity and performance across the life course [90]. From this perspective, functional ability emerges from the interaction between IC and environmental characteristics, providing a multidimensional model for understanding functional trajectories during aging [1,86,91,92,93,94].

Within this framework, IC describes healthy aging from a functional and person-centered perspective, emphasizing the capacities individuals retain rather than the diseases they may develop [90]. IC is defined as the composite of an individual’s physical and mental capacities across the life course, comprising five interconnected domains: locomotion, vitality, sensation, cognition, and psychological function [1,91,93,94]. These domains collectively contribute to autonomy, resilience, and functional independence in older age [92,95].

The preservation and optimization of IC are therefore central to delaying functional decline and supporting autonomy and quality of life in older adults [92,94]. In this context, growing attention has been directed toward nutritional strategies capable of modulating the biological mechanisms implicated in aging-related functional deterioration, including inflammation, oxidative stress, membrane composition, and cellular signaling [1,93,96,97,98]. Bioactive compounds with pleiotropic mechanisms of action may exert system-wide effects that translate into functional benefits across multiple dimensions of IC. Accordingly, the following sections critically examine the mechanistic and clinical evidence regarding the potential role of KO on locomotor, vitality, sensory, cognitive, and psychological functions within the framework of healthy aging.

### 4.2. Domain-Specific Effects of Krill Oil

The multidimensional nature of IC provides a structured framework to evaluate the physiological relevance of nutritional interventions across interconnected domains of function. Within this context, KO has been investigated in a growing number of randomized controlled clinical studies assessing outcomes related to locomotor performance, metabolic vitality, cognitive function, sensory health, and psychological well-being (Table 3). Although the evidence base remains heterogeneous in terms of populations, supplementation protocols, and clinical endpoints, these investigations collectively offer insights into the potential of KO to modulate biological processes associated with aging-related decline. Table 3 summarizes randomized controlled clinical studies conducted in adult and elderly populations that have investigated the effects of oral KO supplementation on outcomes related to IC domains. Because direct intervention studies of KO in healthy older adults remain limited, this section includes evidence from both healthy or community-dwelling populations and patients with established clinical conditions. Findings from healthy populations are considered in the context of health maintenance, whereas studies conducted in patients are interpreted as therapeutic or disease-context evidence and are included primarily to inform biological plausibility and domain-specific responses. Accordingly, findings from pathological populations should not be directly extrapolated to normal aging.

Because KO is a multicomponent nutritional matrix, clinical trials, even when reporting the choline and astaxanthin content of the preparations evaluated, do not permit the independent contributions of phospholipid-bound *n*-3 PUFAs, choline, and astaxanthin to be distinguished. Choline-related findings are discussed below where KO intervention studies directly assessed circulating choline and related metabolites [99,119], whereas the specific contribution of astaxanthin remains biologically plausible but cannot be isolated from the effects of the other constituents. Accordingly, evidence from isolated choline or astaxanthin supplementation was not extrapolated to KO, and their individual contributions within the matrix remain to be established.

A detailed discussion of the molecular and cellular mechanisms underlying these domain-specific effects, including evidence derived from experimental and preclinical studies, is beyond the scope of this review and has been extensively addressed elsewhere [10,17,38,59]. However, to complement the clinical evidence, selected preclinical studies, meta-analyses, and studies in specific populations were included to illustrate potential mechanisms underlying the effects of KO across the domains of IC. Nevertheless, animal data should be interpreted cautiously, as doses cannot be directly extrapolated to humans owing to interspecies differences in metabolism and pharmacokinetics. Therefore, further clinical studies are needed to translate these mechanistic findings into evidence-based KO supplementation strategies for different populations [123].

In addition to the domains discussed in this review, KO has been investigated across a broad range of health applications. The strongest evidence supports its benefits for cardiovascular [18,65,124] and skin health [125], while growing evidence suggests potential roles in liver health [126,127,128], tumor suppression [129], and women’s health [118]. These applications are beyond the scope of the present review and have been comprehensively addressed elsewhere.

**Figure 1:**
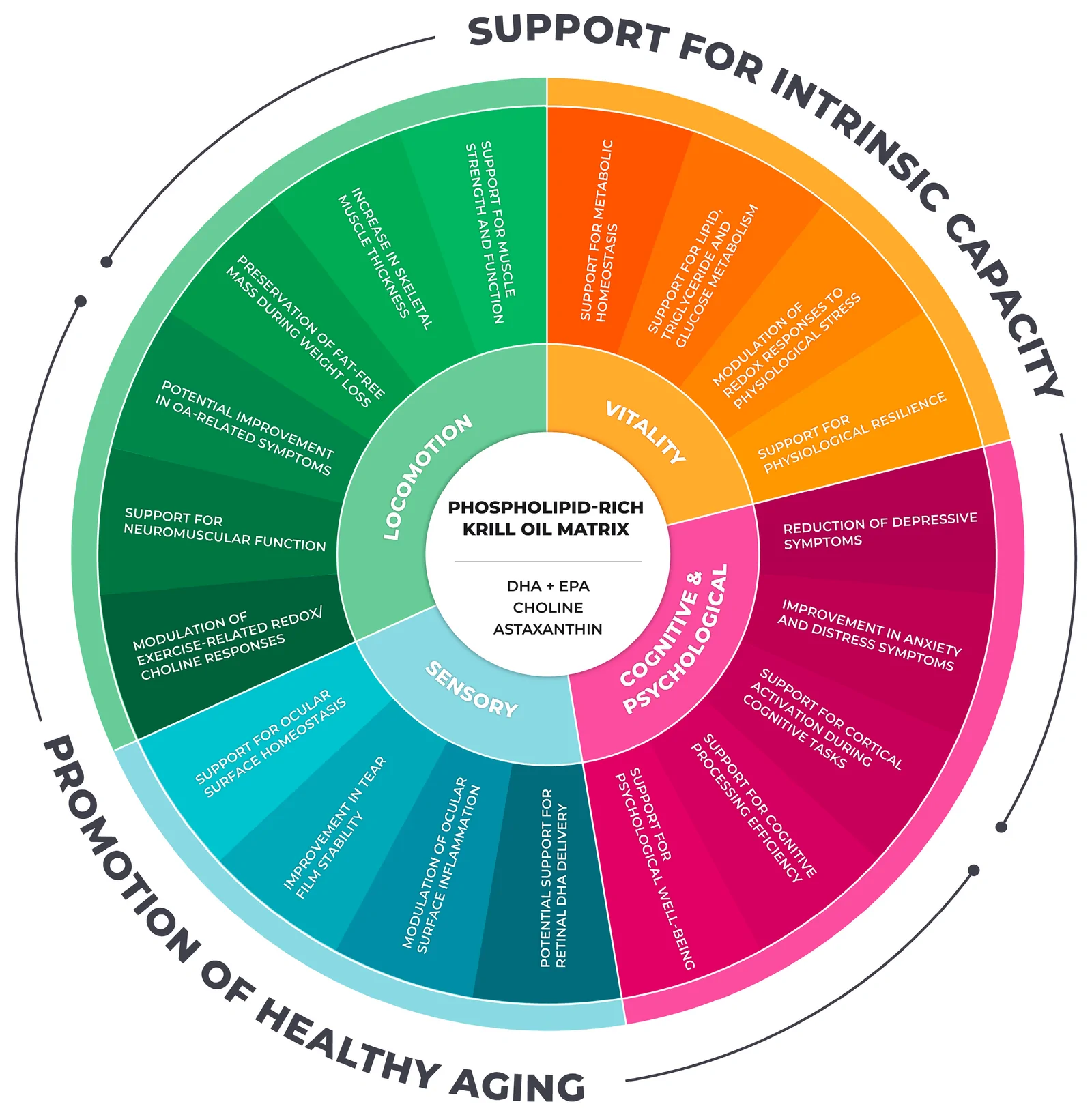
Potential contribution of KO to IC-related domains. KO provides phospholipid-bound EPA and DHA, choline, and astaxanthin, which may influence biological processes relevant to vitality, locomotion, sensory function, and cognitive/psychological capacity.

To complement the clinical evidence summarized in Table 3, Figure 1 provides a conceptual overview of the potential contribution of KO to domains related to IC. Although direct assessment of IC using validated composite instruments has not yet been reported in clinical studies with KO supplementation, the available evidence suggests that its characteristic nutrient matrix, comprising phospholipid-bound EPA and DHA, choline, and astaxanthin, may support multiple physiological functions relevant to healthy aging. These effects have been primarily observed across domains associated with vitality, locomotion, sensory function, and cognitive/psychological capacity, supporting the relevance of KO as a nutritional strategy to promote functional health during aging.

#### 4.2.1. Vitality Domain

Within the IC framework, vitality reflects an individual’s physiological reserve and ability to maintain homeostatic balance in response to internal and external stressors. Vitality is considered a foundational capacity that may influence other IC domains, because aging is characterized by a progressive decline in adaptive homeostasis [130]. It is closely linked to nutritional status, energy balance, and biological aging, and has been assessed using functional measures such as handgrip strength as well as circulating biomarkers associated with inflammation, nutritional status, and physiological resilience [94,131]. Biomarkers including albumin, C-reactive protein (CRP), and hemoglobin may provide integrative insight into metabolic and inflammatory processes relevant to vitality and healthy aging [94].

Collectively, these findings extend beyond conventional lipid management and suggest that improved metabolic homeostasis may contribute to the preservation of physiological reserve, a central component of the vitality domain. By reducing metabolic stress and improving lipid handling, KO may enhance the organism’s capacity to maintain homeostasis in response to age-related physiological challenges. Supporting this notion, a pooled analysis of two phase 3 randomized, double-blind, placebo-controlled trials in patients with severe hypertriglyceridemia showed that supplementation with a KO-derived omega-3 phospholipid/free fatty acid formulation (4 g/day) significantly reduced fasting triglyceride levels by approximately 26% at 12 weeks, with sustained reductions at 26 weeks (*p* = 0.02) and a safety profile comparable to placebo [33]. Similarly, in a multicenter randomized placebo-controlled trial involving adults with borderline high or high triglyceride levels, 12 weeks of KO supplementation was associated with a modest but significant reduction in serum triglycerides relative to placebo (−10.2% vs. placebo-adjusted; *p* = 0.0389), without increasing low-density lipoprotein cholesterol (LDL-C), further supporting a potential role in metabolic homeostasis [62]. However, interpretation of some lipid-lowering findings should remain cautious due to methodological limitations, including pooled dose analyses and substantial interindividual variability in triglyceride responses [62]. Earlier randomized clinical evidence in adults with hyperlipidemia also reported improvements in triglycerides, total cholesterol, LDL-C, high-density lipoprotein cholesterol (HDL-C), and glucose levels following KO supplementation, with some effects exceeding those observed with FO comparators [54].

Mechanistic evidence from a preclinical study in male rats fed a high-cholesterol diet further supports these findings. KO supplementation (100 or 200 mg/kg body weight/day) reduced circulating cholesterol levels by suppressing hepatic cholesterol synthesis through AMP-activated protein kinase (AMPK)-mediated inhibition of 3-hydroxy-3-methyl-glutaryl-coenzyme A (HMG-CoA) reductase, while simultaneously enhancing LDL uptake and cholesterol excretion via increased hepatic LDL receptor expression and fecal elimination of cholesterol and bile acids. These findings provide a plausible biological basis for the lipid-lowering effects observed in clinical studies [132].

In fact, a meta-analysis of seven randomized controlled trials (*n* = 662) further confirmed that KO supplementation could reduce LDL-C and triglycerides, while increasing HDL-C [18]. A more recent meta-analysis of 14 randomized controlled trials (1458 participants) corroborated significant improvements in total cholesterol, LDL-C, and triglycerides, with greater lipid-lowering effects observed in interventions lasting <8 weeks and among individuals with a BMI < 28 kg/m^2^ [65]. In addition to its benefits for the vitality domain, the favorable effects of KO on lipid metabolism may also have broader implications for cardiovascular health, as dyslipidemia is a major modifiable risk factor for cardiovascular disease [133] and early, sustained reductions in low-density lipoprotein cholesterol (LDL-C) have been associated with substantial reductions in lifelong cardiovascular risk [134].

Consistent with a broader metabolic and inflammatory effect, a randomized double-blind crossover trial in mildly overweight adults with hypertriglyceridemia showed that 1000 mg/day of KO for 4 weeks improved triglyceride, HDL-C, apolipoprotein AI, and high-sensitivity C-reactive protein (hs-CRP) levels (*p* < 0.05) [34].

CRP is a key biomarker of inflammaging, the chronic low-grade systemic inflammation that characterizes the aging process and contributes to age-related diseases, frailty, and mortality [135]. Consequently, interventions that attenuate systemic inflammation may help preserve vitality by maintaining metabolic function and reducing cumulative physiological stress. The reduction in hs-CRP observed in this trial supports a potential contribution of KO to metabolic homeostasis beyond triglyceride lowering alone and suggests that KO may contribute to the maintenance of vitality through modulation of age-related inflammatory processes. The anti-inflammatory effects of KO appear to result from the complementary actions of its bioactive constituents, including phospholipid-bound EPA and DHA, which modulate eicosanoid pathways; astaxanthin, which inhibits nuclear factor-kappaB (NF-κB) and mitogen-activated protein kinase (MAPK) signaling; and vitamin E, which contributes to membrane stabilization [136].

Although clinical evidence remains limited, preclinical studies provide mechanistic evidence supporting the metabolic effects of KO. In a mouse model of type 2 diabetes, 6 months of KO supplementation reduced fasting blood glucose, improved insulin resistance, and attenuated white adipose tissue dysfunction [137]. Likewise, in mice with diet-induced obesity, KO phospholipids preserved insulin sensitivity, reduced endogenous glucose production by 47%, and increased whole-body and skeletal muscle glycogen synthesis by approximately threefold relative to high-fat diet controls [138]. Consistent with these findings, the study by Bunea et al. (2004), discussed previously, reported that KO supplementation significantly reduced fasting blood glucose compared with baseline, with greater improvements than FO at equivalent or lower doses [54]. These findings further support the potential role of KO in improving metabolic homeostasis, an important component of the vitality domain.

Additional exploratory evidence supports potential metabolic effects of KO beyond conventional lipid markers. In a pilot randomized trial in adults with familial hypercholesterolemia, KO supplementation (2 g/day) increased erythrocyte DHA levels and the omega-3/-6 ratio, although no significant effects were observed on LDL-C or lipoprotein(a) [64]. However, these findings should be interpreted cautiously, as all participants concomitantly received a dietary intervention (the Brazilian Cardioprotective Diet, DICA-BR) and some groups also received phytosterols, limiting the isolation of KO-specific effects. Similarly, an uncontrolled pilot study in healthy young adults reported improvements in plasma and erythrocyte omega-3 status, antioxidant capacity, and circulating choline-related metabolites following short-term KO supplementation [67]. In a clinical study involving 100 patients with coronary heart disease, KO supplementation increased antioxidant capacity, as demonstrated by higher levels of superoxide dismutase (SOD), glutathione (GSH), and glutathione peroxidase (GPx) compared with the control group [139]. Similarly, in a rat model of dextran sulfate sodium (DSS)-induced colitis, KO demonstrated protective effects against intestinal inflammation, which were associated with reduced oxidative stress markers. Although these findings should be interpreted cautiously due to their preliminary nature and the use of specific populations and preclinical models, they provide supportive mechanistic evidence linking KO to metabolic and redox pathways relevant to physiological resilience.

Complementary evidence for the relationship between KO and vitality suggests effects on biomarkers related to physiological resilience. In a randomized double-blind trial in well-conditioned power-training athletes, 12 weeks of KO supplementation (2.5 g/day, providing 550 mg EPA + DHA and 150 mg choline) increased the HS-Omega-3 Index and reduced the arachidonic acid-to-EPA ratio (AA/EPA ratio, *p* < 0.001), consistent with changes in lipid status and inflammatory balance [99]. The KO group also showed greater recovery of circulating choline between exercise bouts and a smaller post-exercise decline in total antioxidant capacity than the placebo group, suggesting a possible effect on metabolic and redox responses to intense physiological stress [99]. Although conducted in trained athletes and lacking direct clinical endpoints, these findings support the concept that KO may influence mechanisms related to metabolic resilience and recovery.

Taken together, the available clinical and preclinical evidence suggests that KO may support the vitality domain by improving metabolic homeostasis, attenuating low-grade inflammation, enhancing antioxidant defenses, and promoting physiological resilience. Although the individual contributions of phospholipid-bound EPA and DHA, choline, and astaxanthin cannot currently be distinguished in clinical studies, the multimolecular composition of KO provides a biologically plausible rationale for its potential effects on vitality and promote healthy aging. Nevertheless, the relative contribution of each component and the existence of synergistic interactions remain to be established in future mechanistic and clinical studies.

#### 4.2.2. Locomotor Domain

The locomotor domain of IC reflects the integrated function of musculoskeletal and neuromotor systems that underpin mobility, independence, and physical performance during aging. Functional indicators such as gait speed and muscle strength are strongly associated with disability, care dependence, and mortality, and are widely considered as markers of physiological reserve and biological aging [140,141,142,143,144]. Age-related impairments in locomotor capacity are closely linked to chronic inflammation, reduced aerobic capacity, and cardiometabolic dysfunction, reinforcing the relevance of this domain as a target for interventions aimed at preserving functional ability in older adults [145,146,147].

Osteoarthritis (OA), a major contributor to locomotor disability and chronic pain in aging populations, has been one of the main clinical contexts in which KO has been investigated [61]. Pre-clinical and clinical evidence indicates that KO may improve OA-associated knee symptoms [30,61,103]. Supporting mechanistic evidence comes from a preclinical study in male rats with monosodium iodoacetate (MIA)-induced osteoarthritis. KO supplementation (100 or 150 mg/kg body weight/day) protected against cartilage degeneration by suppressing inflammatory mediators, including prostaglandin E2 (PGE_2_), interleukin-1beta (IL-1β), tumor necrosis factor-alpha (TNF-α), cyclooxygenase-2 (COX-2), and NF-κB, while reducing matrix metalloproteinase expression, and preserved extracellular matrix components, including aggrecan and collagen. These findings suggest that KO may alleviate OA symptoms through complementary anti-inflammatory and chondroprotective mechanisms [148]. In a subsequent MIA-induced osteoarthritis model, supplementation with 150 mg/kg body weight/day also improved locomotor performance, as demonstrated by increased running speed, stride length, and foot pressure. Complementary *in vitro* experiments further showed that KO protected chondrocytes and synovial cells against oxidative and inflammatory injury by suppressing apoptosis and matrix degradation, while enhancing the expression of anabolic cartilage markers, such as aggrecan, collagen types I and II, tissue inhibitor of metalloprotease-1 (TIMP-1), and TIMP-3 and reducing catabolic mediators, including matrix metalloproteinase-3 (MMP-3), matrix metalloproteinase-13 (MMP-13), and phosphorylated Smad [149]. Together, these findings provide a plausible biological basis for the beneficial effects of KO on OA symptoms.

Suzuki et al. (2016) reported in a randomized, placebo-controlled trial involving 50 adults with mild knee pain that KO (2 g/day; 0.35 g/day EPA + DHA) led to greater improvements compared with placebo in selected symptoms related to pain and stiffness, as assessed by the Japanese Knee Osteoarthritis Measure and Japanese Orthopaedic Association score, although both groups showed overall improvements across multiple questionnaire items [103]. Similarly, Deutsch et al. (2007), in a randomized, double-blind, placebo-controlled trial in 90 patients with cardiovascular disease and/or rheumatoid arthritis and/or OA and elevated C-reactive protein (CRP) levels, demonstrating that KO (300 mg/day) significantly reduced CRP concentrations as early as 7 days, with further reductions at 14 and 30 days (*p* = 0.049, 0.004, and 0.008, respectively), alongside significant improvements in Western Ontario and McMaster Universities Osteoarthritis Index (WOMAC) pain (*p* = 0.050), stiffness (*p* = 0.001), and physical function (*p* = 0.008) scores compared with placebo [30]. More recently, Stonehouse et al. (2022), in a multicenter, randomized, double-blind, placebo-controlled trial involving 235 adults with mild to moderate knee OA, showed that KO supplementation over 6 months resulted in modest but statistically significant improvements in knee pain, stiffness, and physical function compared with placebo (*p* < 0.05), while demonstrating a favorable safety profile [61].

However, subsequent evidence from a larger multicenter randomized clinical trial has tempered these findings [104]. In 262 adults with symptomatic knee OA and magnetic resonance imaging (MRI)-detected effusion-synovitis, 24 weeks of KO supplementation (2 g/day) did not improve knee pain, WOMAC scores, lower-limb strength, or most secondary outcomes compared with placebo. Moreover, reductions in effusion-synovitis volume were greater in the placebo group [104]. Despite the negative findings reported by Laslett et al. [104], a recent Bayesian network meta-analysis including 35 randomized controlled trials (*n* = 4253) comparing several nutritional supplements for knee osteoarthritis identified KO as one of the most promising interventions for improving joint function and stiffness [150]. Based on SUCRA rankings, KO ranked second only to *Boswellia* for WOMAC stiffness and function, whereas its effect on pain was more modest. These findings suggest that the clinical benefits of KO may be more consistently reflected in functional outcomes than in pain relief alone, although additional high-quality trials are needed to clarify its therapeutic role [150]. These findings suggest that the clinical benefits of KO may be more consistently reflected in functional performance than pain relief alone. Nevertheless, the heterogeneity among studies highlights the need for additional well-designed trials to identify the patient populations and clinical settings most likely to benefit from KO supplementation.

Beyond joint-related outcomes, emerging clinical evidence suggests that KO may also influence skeletal muscle mass and function. This is particularly relevant because age-related loss of skeletal muscle mass and strength (sarcopenia) is an important determinant of declining locomotor capacity, contributing to impaired mobility, frailty, falls, loss of independence, and disability in older adults [151]. Sarcopenia is a multifactorial condition driven by chronic low-grade inflammation, anabolic resistance, impaired protein turnover, and metabolic dysfunction, all of which contribute to the progressive decline in muscle mass and strength during aging [151]. In addition to its anti-inflammatory properties (Section 4.2.1. Vitality), KO may promote skeletal muscle anabolism through modulation of key pathways involved in protein metabolism. In cultured skeletal muscle cells, KO activated mTOR signaling across all tested concentrations, suggesting a potential role in stimulating muscle protein synthesis [152]. More recently, preclinical evidence in a mouse model of type 2 diabetes-induced sarcopenia demonstrated that long-term dietary KO supplementation preserved skeletal muscle mass and strength, improved insulin sensitivity, reduced oxidative stress and pro-inflammatory cytokine production, and restored protein homeostasis by activating the PI3K/Akt/mTOR pathway while suppressing FoxO3a- and NF-κB-mediated protein degradation [123].

In a double-blind randomized controlled trial in healthy adults, 6 months of KO supplementation (4 g/day) significantly increased knee extensor maximal torque, grip strength (10.9%), and vastus lateralis muscle thickness (3.5%) compared with the control (*p* < 0.05), indicating favorable effects on muscle performance and morphology [102]. A subsequent exploratory subgroup analysis suggested that these effects were generally consistent across sex, age, and BMI categories, although some neuromuscular effects appeared more pronounced in men [100]. In adults with overweight or obesity undergoing alternate-day fasting, KO supplementation attenuated the loss of fat-free mass and handgrip strength during weight loss, supporting a potential role in preserving muscle-related functional parameters under catabolic conditions [101]. Collectively, these findings extend the relevance of KO within the locomotor domain beyond symptom management in OA.

KO may also influence locomotor function through modulation of oxidative stress and recovery responses associated with muscle exertion. In elite rowers, supplementation with KO (1 g/day, 6 weeks) attenuated post-exercise oxidative damage during recovery, although no effects were observed on antioxidant enzymes or TNF-α concentrations [120]. While conducted in highly trained athletes and lacking functional outcomes, these findings suggest that KO may contribute to redox homeostasis under conditions of physiological stress, a mechanism that may also be relevant to age-related decline in physical performance.

Choline may also contribute to locomotor capacity through its role in neuromuscular function and membrane homeostasis. As a precursor of acetylcholine and phosphatidylcholine, choline is essential for skeletal muscle contraction and membrane integrity, and circulating levels may decline during prolonged and high-intensity exertion [119,153,154]. In an exploratory study in endurance athletes, KO supplementation (4 g/day, 5 weeks) increased circulating choline and its related metabolites and attenuated exercise-induced declines in serum choline [119]. While these findings remain preliminary and were not associated with direct functional outcomes, they provide mechanistic support for a potential contribution of phospholipid-bound choline to neuromuscular function and locomotor resilience.

Overall, the available evidence suggests that KO may support the locomotor domain of intrinsic capacity through complementary effects on joint symptoms, skeletal muscle function and metabolism, inflammatory balance, oxidative stress, and neuromuscular metabolism. Although the evidence for osteoarthritis remains heterogeneous and additional high-quality trials in older adults are warranted, the multimolecular composition of KO provides a biologically plausible rationale for its potential to preserve mobility, muscle function, and physical independence during aging.

#### 4.2.3. Cognitive and Psychological Domains

The cognitive and psychological domains of IC are key determinants of functional independence and adaptive capacity during aging. Cognitive function shows substantial interindividual variability, with accelerated decline associated with increased risk of adverse health outcomes and loss of autonomy, and it interacts closely with other domains of IC [91,155,156]. In parallel, psychological capacity, particularly mood and emotional regulation, is strongly linked to functional status, as depressive symptoms are associated with increased vulnerability to physical decline and adverse health outcomes [91,157]. Together, these domains represent interconnected dimensions of IC that may be influenced by nutritional, metabolic, and neurobiological factors relevant to healthy aging.

Given its unique combination of phospholipid-bound EPA and DHA, astaxanthin, and choline, KO may modulate multiple mechanisms involved in age-related neuroinflammation, including oxidative stress, inflammatory signaling, and neuronal membrane integrity, thereby supporting the preservation of cognitive capacity during aging [4,158]. These mechanistic observations are supported by experimental models, in which KO consistently improved learning, memory, and recognition performance while attenuating oxidative stress, suggesting that modulation of neuroinflammation and redox homeostasis may underlie its neuroprotective effects. In senescence-accelerated mice, KO improved learning and memory deficits in Morris water maze and Barnes maze tests [159]. In high-fat diet-induced cognitive impairment models, KO administration (100–500 mg/kg/day for 4 weeks) improved spatial memory, novel object recognition, and learning abilities [160]. Additional mechanistic evidence has been obtained in an amyloid-β25-35 (Aβ25-35)-induced mouse model of Alzheimer’s disease. KO supplementation significantly improved recognition memory, learning, and spatial memory while reducing neuronal oxidative damage, as evidenced by lower brain levels of reactive oxygen species (ROS), malondialdehyde (MDA), and nitric oxide (NO). KO also attenuated neuronal apoptosis, indicating preservation of neuronal survival pathways. Together, these findings suggest that KO may counteract key pathological processes underlying Alzheimer’s disease, including oxidative stress, apoptosis, and neuroinflammation, providing a biologically plausible basis for its potential role in preserving cognitive capacity during aging [161].

In a randomized controlled trial in healthy older adults, KO supplementation over 12 weeks was associated with enhanced cerebral cortical activation during cognitive tasks, assessed by near-infrared spectroscopy, and improved electrophysiological markers of cognitive processing (reduced P300 latency), compared to placebo (*p* = 0.03) [56]. Notably, these effects were more pronounced than those observed with triglyceride-based FO, raising the possibility that lipid structure may influence neurophysiological responses to omega-3 supplementation.

By contrast, a large randomized, double-blind, placebo-controlled trial involving 245 young military officers found no effects of KO supplementation on cognitive performance, resilience, or mood after 20 weeks of intervention [105]. Interpretation of these findings should consider the relatively young, healthy, and highly trained study population, which may have limited the detection of additional benefits, as well as the high dropout rate (55.9%) and suboptimal adherence during the study [105].

Beyond cognition, preservation of psychological capacity is another essential component of intrinsic capacity, as depressive symptoms are associated with accelerated functional decline and reduced resilience during aging [162]. In a randomized, double-blind, placebo-controlled trial in adults with major depressive disorder, 8 weeks of KO supplementation was associated with significant reductions in depressive symptoms compared with placebo, as reflected by decreases in Hamilton Depression Rating Scale scores and a significant group-by-time interaction [55].

In preclinical models of chronic unpredictable mild stress-induced depression, KO ameliorated depression-like behaviors through restoration of glycerophospholipid and sphingolipid metabolism, with effects mediated through membrane structure modulation and neuroactive ligand–receptor interaction pathways [163]. Animal studies also demonstrated reduced anxiety in senescence-accelerated mice following KO treatment [159]. The underlying mechanisms supporting the potential effects of KO on the psychological domain have been further explored in experimental models of brain aging. In a D-galactose-induced aging mouse model, KO attenuated oxidative stress, enhanced the expression of antioxidant enzymes, and modulated proteins involved in dopaminergic neurotransmission, including increased expression of tyrosine hydroxylase (TH) and aromatic L-amino acid decarboxylase (AADC), two key enzymes required for dopamine synthesis, while also promoting gut microbiota changes characterized by increased abundance of *Lactobacillus* spp. and short-chain fatty acid-producing bacteria [164]. These findings are mechanistically relevant because aging and chronic low-grade inflammation impair dopaminergic signaling by reducing dopamine synthesis, availability, and receptor function, which contributes to deficits in cognition, motivation, motor performance, and emotional regulation [165]. As these interconnected processes are recognized contributors to late-life depression [165], the preservation of dopaminergic function by KO provides a plausible biological mechanism linking its antioxidant and anti-inflammatory properties to the maintenance of psychological capacity during aging.

Taken together, the available evidence suggests that KO may support both cognitive and psychological domains of intrinsic capacity through complementary mechanisms involving neuronal membrane integrity, neuroinflammation, oxidative stress, and lipid-mediated signaling. Nevertheless, the current clinical evidence remains limited and heterogeneous, warranting larger randomized trials in older adults.

#### 4.2.4. Sensory Domain

The sensory domain of IC encompasses visual and auditory functions that are critical for maintaining independence, communication, and social participation during aging. Sensory impairments are strongly associated with functional decline, reduced quality of life, and increased risk of disability, particularly when visual and auditory deficits coexist [91,166,167]. Among sensory conditions relevant to aging, dry eye disease is particularly common and clinically meaningful, with substantial impact on visual comfort and quality of life. It has been estimated that approximately 68% of individuals aged 60 years or older report dry eye-related symptoms, including burning, foreign body sensation, visual discomfort, and ocular fatigue [168].

Clinical evidence suggests that KO may influence ocular surface function and inflammatory processes relevant to sensory capacity [169,170]. In a randomized, double-masked, placebo-controlled clinical trial in adults with mild to moderate dry eye disease, 90 days of KO supplementation improved tear film stability, reduced tear osmolarity and ocular bulbar redness, and significantly decreased Ocular Surface Disease Index scores relative to placebo. KO supplementation significantly decreased basal tear levels of interleukin-17A, a pro-inflammatory cytokine implicated in ocular surface inflammation [63]. Although conducted in a disease-specific population rather than in older adults, these findings provide preliminary evidence that KO may support sensory function through modulation of ocular surface homeostasis and inflammatory pathways.

A broader mechanistic rationale for potential sensory effects is also supported by the biology of retinal omega-3 metabolism. The retina contains the highest concentration of DHA in the body and depends substantially on dietary supply of this fatty acid to maintain photoreceptor function and structure [171,172]. Experimental evidence further suggests that phospholipid-rich KO may facilitate retinal delivery of *n*-3 PUFAs more efficiently than conventional FO, which is biologically relevant given the role of DHA in modulating inflammation, oxidative stress, and retinal vulnerability [172].

Importantly, direct clinical evidence within the sensory domain remains limited to a single study [63], precluding broader conclusions regarding sensory function or health maintenance during aging. Further randomized controlled trials are needed to determine whether KO exerts clinically meaningful effects across this domain.

## 5. Conclusions and Future Perspectives

KO is a marine-derived nutritional ingredient with a distinctive biochemical profile that differentiates it from conventional FO. Its phospholipid-rich matrix, along with endogenous EPA, DHA, choline, and astaxanthin, provides a biologically plausible rationale for effects on membrane remodeling, inflammatory signaling, redox homeostasis, lipid metabolism, and neuromuscular and neuronal function. Accordingly, current evidence suggest that KO may influence several physiological systems relevant to healthy aging and IC, particularly locomotor function, metabolic vitality, ocular surface homeostasis, and cognitive/psychological processes. To our knowledge, this is the first review to integrate the available evidence on KO within the WHO IC framework, highlighting how a single nutritional intervention may simultaneously influence multiple domains that collectively determine functional ability during aging.

Clinical studies have reported improvements in selected outcomes related to knee pain and function, skeletal muscle strength and thickness, triglyceride metabolism, dry eye parameters, cerebral activation during cognitive tasks, and depressive symptoms. However, the available evidence remains heterogeneous owing to differences in study populations, supplementation doses, KO formulations, comparators, intervention duration, and outcome measures. Furthermore, many studies were conducted in patients with established diseases, whose metabolic and physiological responses may differ from those observed during healthy aging. Therefore, these findings should be interpreted as complementary evidence supporting the relevance of KO to specific IC-related domains rather than as direct evidence of IC in healthy older adults.

An additional limitation is that none of the currently available clinical trials were specifically designed to evaluate IC as an integrated construct. Instead, evidence remains fragmented across isolated physiological outcomes, precluding conclusions regarding the overall preservation of functional ability during aging. Moreover, most available studies have evaluated short-term surrogate outcomes, whereas longitudinal changes in IC occur progressively over years. Consequently, it remains uncertain whether the domain-specific improvements reported to date translate into slower trajectories of functional decline or reduced risk of frailty and disability.

Future randomized controlled trials should therefore adopt multidomain study designs enrolling middle-aged and older adults across different stages of functional decline, including robust, prefrail, and frail individuals. Longitudinal follow-up is particularly important to determine whether KO supplementation modifies the trajectory of age-related decline in IC rather than only producing short-term physiological improvements. Whenever possible, IC should be assessed using standardized multidomain instruments, such as the WHO Integrated Care for Older People (ICOPE) screening pathway, which has demonstrated high sensitivity (95%) and specificity for detecting declines in intrinsic capacity [173,174], or validated composite scoring systems that quantify each IC domain and generate an overall IC score, such as the approach proposed by López-Ortiz et al. (2022) [122]. These integrated assessments should be complemented by clinically meaningful functional endpoints together with biomarkers of omega-3 status, inflammation, oxidative stress, membrane lipid remodeling, and choline metabolism, thereby facilitating the identification of biological mechanisms linking KO supplementation to changes in IC.

Another important methodological consideration is the need for greater standardization across future clinical studies. Given the profound physiological changes accompanying aging, participants should be stratified according to age and functional status to distinguish the effects of KO from those attributable to normal aging alone. Such methodological advancement will be essential to identify the populations most likely to benefit from KO supplementation and to determine whether KO is more effective as an early preventive nutritional strategy aimed at preserving intrinsic capacity rather than as an intervention after substantial functional decline has already occurred.

Overall, the strongest clinical support currently relates to locomotor and vitality-related outcomes, especially skeletal muscle function in older adults and cardiometabolic markers in subjects with hypertriglyceridemia. In contrast, evidence for the sensory and cognitive/psychological domains remains promising but preliminary, being derived primarily from disease-specific populations or relatively small clinical trials. Accordingly, while future multidomain studies assessing IC as an integrated construct are warranted, additional well-designed randomized controlled trials focusing on individual IC domains are also needed to strengthen the evidence base, particularly for sensory, cognitive, and psychological functions, where clinical data remain scarce.

At present, no international guideline establishes a KO-specific intake recommendation for older adults. Existing recommendations are expressed as combined EPA + DHA, irrespective of source. The European Food Safety Authority (EFSA) recommends an adequate intake of 250 mg/day of EPA + DHA for the general adult population [175], whereas the International Society for the Study of Fatty Acids and Lipids (ISSFAL) recommends ≥500 mg/day [176]. Additionally, the American Heart Association (AHA) recommends 1 g/day of EPA + DHA for secondary cardiovascular prevention [177,178]. In the limited clinical trials conducted in healthy older adults, KO doses of 2–4 g/day, providing approximately 0.29–1.16 g/day of EPA + DHA, were administered for 3–6 months [56,100,102]. However, current evidence remains insufficient to establish an optimal KO intake for preserving IC during healthy aging.

The concept of IC emphasizes that healthy aging depends on preserving multiple interconnected physiological functions rather than treating individual diseases. Within this framework, KO is particularly attractive because its multimolecular composition targets several complementary biological pathways simultaneously, supporting a systems-based approach to nutritional interventions for healthy aging.

In conclusion, the available evidence suggests that KO has the potential to support healthy aging through complementary mechanisms spanning multiple domains of IC, including metabolic homeostasis, inflammation, oxidative stress, musculoskeletal function, neuronal signaling, and ocular surface physiology. Although definitive conclusions await larger, longer-term, and methodologically standardized randomized clinical trials, the current evidence provides a strong biological rationale for considering KO as a promising nutritional strategy to preserve IC, delay functional decline, and promote healthy longevity.

## Figures and Tables

**Table 1:** Reported ranges of the major bioactive constituents in KO according to different literature sources. Variations in composition reflect differences in extraction methods, processing conditions, and the natural biological variability of krill. Therefore, the table summarizes the principal bioactive constituents reported in representative studies that are most relevant from nutritional and physiological perspectives. The values represent the mean or reported range of concentrations from the respective studies, and the corresponding units of measurement are indicated in the table.

Component	Unit	Kwantes et al., 2015 [17]	Xie et al., 2017 [27]	Xie et al., 2018 [28]	Xie et al., 2019 [25]	Kutzner et al., 2017 [29]
EPA (20:5 *n*-3)	% total FA	14	12.61–17.81	9.09–24.81	14.3–28.0	10.3–11.9
DHA (22:6 *n*-3)	% total FA	6.5	8.78–12.32	4.72–13.87	7.1–15.7	5.2–5.8
DPA (22:5 *n*-3)	% total FA	NA	0.41–0.49	0.31–0.56	ND–0.07	0.26–0.29
ALA (18:3 *n*-3)	% total FA	NA	1.28–2.39	2.14–2.34	0.1–4.7	0.97–1.83
∑SFA	% total FA	NA	34.42–37.56	32.69–41.28	NA	18.8–19.6
∑MUFA	% total FA	NA	29.49–35.14	22.89–39.34	NA	12.9–15.3
∑PUFA	% total FA	NA	27.36–35.84	19.38–44.42	NA	19.0–19.9
∑PUFA *n*-3	% total FA	NA	24.24–33.05	16.63–41.74	NA	17.2–18.7
Astaxanthin	mg/kg	NA	93.87–222.64	7.48–505.00	40–5000	40–5000

Abbreviations: ALA, alpha-linolenic acid; DHA, docosahexaenoic acid; DPA, docosapentaenoic acid; FA, fatty acid; EPA, eicosapentaenoic acid; NA: not applicable or available; ND: not detected; ∑SFA, total saturated fatty acids, ∑MUFA, total monounsaturated fatty acids; ∑PUFA, total polyunsaturated fatty acids; ∑PUFA *n*-3, total omega-3 polyunsaturated fatty acids.

**Table 2:** Key compositional and nutritional differences between KO and conventional FO.

Aspect	Comparative Evidence	Interpretation	References
Nutrient matrix	In KO, approximately 30–65% of fatty acids are esterified to phospholipids, mainly phosphatidylcholine, together with choline and astaxanthin. FO provides EPA and DHA mainly as triacylglycerols.	KO provides a distinct phospholipid-rich, multicomponent nutrient matrix.	[4,8,10,17,18,25,26]
Blood EPA and DHA response	KO produced a similar increase in plasma EPA and DHA despite providing 543 vs. 864 mg/day EPA + DHA with FO. At matched total *n*-3 PUFA doses, KO produced greater increases in plasma and erythrocyte EPA and total *n*-3 PUFAs and in Omega-3 Index. At an equal dose of 1.1 g/day *n*-3 PUFAs, KO produced ~1.5-fold greater increases in plasma EPA and DHA. Other matched-dose trials found comparable blood EPA + DHA responses.	KO may offer greater omega-3 dose efficiency under selected conditions, although findings are not uniform.	[8,40,47,49,50]
Lipid distribution and absorption kinetics	KO-derived EPA and DHA were preferentially increased in plasma phospholipid and ether-phospholipid fractions, whereas FO fatty acids were more prominent in triglyceride-rich fractions. Phospholipid-enhanced FO produced an earlier and greater EPA peak than KO.	Lipid form and formulation influence postabsorptive handling; phospholipid content may be more relevant than marine source alone.	[48,52,53]
Comparative clinical outcomes	KO showed greater improvements in HDL-C, ApoAI, and hs-CRP than omega-3 ethyl esters, whereas ethyl esters produced greater TG lowering. Compared to FO, KO showed greater reductions in glucose and LDL-C and, at higher doses, TGs. Both oils improved depressive symptoms, while KO showed significant effects across more neurophysiological outcomes than sardine oil.	Potential advantages of KO are outcome-specific; consistent clinical superiority over FO has not been established.	[34,54,55,56]

Abbreviations: ApoAI, apolipoprotein AI; DHA, docosahexaenoic acid; EE, ethyl ester; EPA, eicosapentaenoic acid; FO, fish oil; HDL-C, high-density lipoprotein cholesterol; hs-CRP, high-sensitivity C-reactive protein; KO, krill oil; *n*-3 PUFAs, omega-3 polyunsaturated fatty acids; TG, triglyceride.

**Table 3:** Randomized, double-blind, controlled clinical trials in adults and elderly people that have evaluated the potential effects of oral KO supplementation on IC-related outcomes.

Reference *	Participant Characteristics	Intervention	IC Domain ^#^	Main Findings
Mozaffarian et al., 2022 [33]	Adults with severe hypertriglyceridemia (fasting TG 500–1500 mg/dL)/54.9 ± 11.2 y./65.2% male/mean BMI 31.5 ± 5.1 kg/m^2^	KO 4 g/day (1.24 g EPA + DHA; *n* = 372) vs. placebo (cornstarch; *n* = 148) for 26 weeks.	Vitality (metabolic health; lipid metabolism—TG and lipoproteins).	KO reduced TG levels vs. placebo at 12 and 26 weeks and modestly reduced non-HDL-C at 26 weeks.
Drobnic et al., 2021 [99]	Healthy, well-conditioned power training athletes (adults)/KO: 32.5 ± 9.6 y; 84.2% male; mean BMI 23.9 ± 2.4 kg/m^2^/placebo: 33.7 ± 8.0 y; 68.8% male; BMI 24.3 ± 2.7 kg/m^2^.	KO 2.5 g/day (Neptune krill oil™; 550 mg EPA + DHA, 150 mg choline and 691 mg/kg esterified astaxanthin; *n* = 19) vs. placebo (virgin olive oil; *n* = 16) for 12 weeks.	Vitality (primary); locomotor (secondary mechanistic relevance to locomotor function).	KO increased the HS-Omega-3 Index, reduced the AA/EPA ratio, enhanced post-exercise plasma choline recovery, and attenuated the decline in antioxidant capacity.
Cicero et al., 2016 [34]	Mildly overweight adults with moderate hypertriglyceridemia (TG 150–500 mg/dL)/KO: 51.1 ± 6.5; 53.8% male; mean BMI 27.9 ± 1.2 kg/m^2^/50.3 ± 5.6 y; 58.3% male; mean BMI 27.6 ± 1.7 kg/m^2^.	KO 1 g/day (750 mg EPA and 90 mg DHA; *n* = 13) vs. 2000 mg/day *n*-3 ethyl esters (at least 85% EPA + DHA, EPA/DHA ratio 0.9–1.5, *n* = 12) for 4 weeks each with 4-week washout.	Vitality (metabolic health, lipid metabolism and inflammation)	Both treatments reduced TG and hs-CRP. KO improved HDL-C, non-HDL-C, and ApoAI, whereas *n*-3ethyl esters produced greater TG reduction.
Berge et al., 2014 [62]	Adults with borderline high or high TG (150–499 mg/dL)/21–79 y/69% male/mean BMI 29.6 ± 3 kg/m^2^.	KO 0.5 g/day (*n* = 53), 1 g/day (*n* = 53), 2 g/day (*n* = 51), or 4 g/day (*n* = 58) (Superba™; 100, 200, 400, and 800 mg EPA + DHA, respectively) vs. placebo (olive oil, *n* = 52) for 12 weeks.	Vitality (cardiometabolic function: lipid metabolism)	KO reduced TG without increasing LDL-C and increased the *n*-3 Index across all doses.
Bunea et al., 2004 [54]	Adults with hyperlipidemia/25–75 y/25% of participants had obesity (BMI ≥ 30 kg/m^2^), 53% were overweight (BMI 25.0–29.9 kg/m^2^), and 22% had a BMI ≤25 kg/m^2^.	KO 1–1.5 g/day or 2–3 g/day, BMI-dependent (Neptune Krill Oil™, *n* = 60) vs. FO 3 g/day (*n* = 30) or placebo (*n* = 30) for 12 weeks.	Vitality (metabolic health; lipid metabolism and glucose).	KO improved lipid profile and glucose, with greater effects than FO or placebo. TG reductions was significant at 2–3 g/day.
Hayman et al., 2026 [100]	Healthy older adults/≥65 y/43.6% male/BMI < 35 kg/m^2^.	KO 4 g/day (SuperbaBoost™; 772 mg EPA, 384 mg DHA, and 316 mg choline, *n* = 49) vs. control 4 g/day (mixed vegetable oil–olive, maize, palm kernel, and MCT oils in a 4:4:3:2 ratio, *n* = 45) for 6 mo.	Locomotor (muscle strength, size and physical function)	KO increased muscle strength, vastus lateralis thickness, and neuromuscular function.
Alblaji et al., 2025 [101]	Adults with overweight/obesity/39 ± 10 y/39% male/mean BMI 31.1 ± 4.2 kg/m^2^.	KO (764 mg EPA, 376 mg DHA, 312 mg choline, and 400 mcg astaxanthin, *n* = 21) vs. placebo (vegetable oil, *n* = 20) during 8 weeks of alternate-day fasting.	Locomotor (muscle function and preservation of fat-free mass during weight loss).	KO preserved fat-free mass and handgrip strength during weight loss.
Stonehouse et al., 2022 [61]	Healthy adults, BMI 18.5–35 kg/m^2^, with mild to moderate knee OA/40–65 y/45.1% male.	KO 4 g/day (SuperbaBoost™; 600 mg EPA, 280 mg DHA, 0.45 mg astaxanthin, *n* = 117) vs. placebo (mixed vegetable oil, *n* = 118) for 6 mo.	Locomotor (knee pain, stiffness, physical function assessed by WOMAC).	KO increased the *n*-3 Index and WOMAC knee pain, stiffness, and physical function.
Alkhedhairi et al., 2022 [102]	Healthy older adults with BMI < 35 kg/m^2^ and <1 h/week of structured exercise/71.2 ± 5.1 y/43.6% male.	KO 4 g/day (SuperbaBoost™; 772 mg EPA, 384 mg DHA, 316 mg choline, *n* = 49) vs. placebo (mixed vegetable oil, *n* = 45) for 6 mo.	Locomotor (skeletal muscle function: knee extensor strength, grip strength, muscle thickness).	KO improved muscle strength and vastus lateralis muscle thickness.
Suzuki et al., 2016 [103]	Adults with mild knee pain/38–85 y/12.7% male.	KO 2 g/day (Superba™; 240 mg EPA and 110 mg DHA, *n* = 25) vs. placebo (safflower oil, *n* = 22) for 30 days.	Locomotor (joint pain, stiffness, and range of motion).	KO improved knee pain, range of motion, JKOM scores, plasma EPA, and EPA/AA ratio.
Deutsch, 2007 [30]	Adults with chronic inflammation (CRP ≥ 1.0 mg/dL) and cardiovascular disease and/or rheumatoid arthritis and/or OA/KO: 54.6 ± 14.8; 55.6% male/55.3 ± 14.3 y; 48.5% male.	KO 300 mg/day (Neptune Krill Oil™; 17% EPA and 10% DHA, *n* = 45) vs. placebo (microcrystalline cellulose, *n* = 45) for 30 days.	Locomotor (arthritic symptoms: pain, stiffness, physical function assessed by WOMAC; systemic inflammation as mechanistic support)	KO reduced CRP and improved WOMAC pain, stiffness, and physical function, with early effects after 7 days.
Laslett et al., 2024 [104]	Adults with symptomatic knee OA and effusion-synovitis/KO: 61.4 ± 9.9 y; 51% male; mean BMI 29.3 ± 5.6 kg/m^2^/placebo: 61.7 ± 9.3 y; 42% male; BMI 29.9 ± 6.5 kg/m^2^.	KO 2 g/day (380 mg EPA, 200 mg DHA, *n* = 130) vs. placebo (vegetable oil blend, *n* = 132) for 24 weeks.	Locomotor (knee pain, physical function and strength).	No significant effects on knee pain, WOMAC outcomes, or lower limb strength vs. placebo.
Açik et al., 2025 [55]	Adults with MDD/*n* = 50/36.1 ± 12.7 y/28% male.	KO 2 g/day (340 mg EPA, 180 mg DHA, *n* = 17) or FO (360 mg EPA, 240 mg DHA, *n* = 17) vs. placebo (soybean oil, *n* = 16) for 8 weeks.	Psychological (depression, anxiety, and distress assessed by DASS-21 and HDRS).	Both KO and FO reduced depression, anxiety, and distress scores. KO reduced HbA1c and TG levels and increased HDL-C.
Konagai et al., 2013 [56]	Healthy elderly males/100% male/KO: 67.1 ± 3.7 y; mean BMI 22.8 ± 2.6 kg/m^2^/sardine oil: 67.0 ± 3.3 y; BMI 23.6 ± 2.2 kg/m^2/^placebo: 67.3 ± 3.4 y; BMI 23.7 ± 3.5 kg/m^2^.	KO 2 g/day (193 mg EPA and 92 mg DHA, *n* = 13) vs. sardine oil 2 g/day (491 mg EPA and 251 mg DHA, *n* = 14) vs. placebo (MCT, *n* = 15) for 12 weeks.	Cognition/psychological (psychophysiological function; working memory, calculation tasks).	KO increased cerebral O_2_Hb during cognitive tasks and reduced P300 latency, with effects comparable to or greater than FO.
Marriott et al., 2021 [105]	Young healthy US Army military officers/23.4 ± 2.8 y.	KO capsules providing 2.3 g/day *n*-3 (EPA/DHA ratio 2:1, *n* = 130) vs. placebo (macadamia nut oil, *n* = 115) for 20 weeks.	Cognition/psychological (cognitive performance, resilience, mood).	KO did not improve cognitive performance, resilience, or mood vs. control.
Deinema et al., 2017 [63]	Adults with mild to moderate dry eye disease/KO: 42.3 ± 3.8 y, 28% male; FO: 39.4 ± 3.4 y, 53% male; placebo: 46.2 ± 4.5 y, 18% male.	KO (945 mg EPA and 510 mg DHA, *n* = 18) vs. FO (1000 mg EPA and 500 mg DHA, *n* = 19) vs. placebo (olive oil, *n* = 17) for 90 days.	Sensory (ocular function; dry eye disease)	KO reduced tear osmolarity and improved dry eye symptoms, tear stability, and ocular redness.

Abbreviations: AA, arachidonic acid; ApoAI, apolipoprotein AI; BMI, body mass index; CRP, C-reactive protein; DASS-21, Depression Anxiety Stress Scales-21; DHA, docosahexaenoic acid; EPA, eicosapentaenoic acid; FO, fish oil; GLM, generalized linear model; HbA1c, glycated hemoglobin; HDL-C, high-density lipoprotein cholesterol; HDRS, Hamilton Depression Rating Scale; IC, intrinsic capacity; IL-17A, interleukin-17A; JKOM, Japanese Knee Osteoarthritis Measure; KO, krill oil; LDL-C, low-density lipoprotein cholesterol; MCT, medium-chain triglycerides; MDD, major depressive disorder; mo, months; *n*-3, omega-3; OA, osteoarthritis; O_2_Hb, oxyhemoglobin; OSDI, Ocular Surface Disease Index; TG, triglyceride; vs, versus; WOMAC, Western Ontario and McMaster Universities Osteoarthritis Index; y, years. * Only randomized controlled studies conducted in adult or older populations and evaluating oral KO supplementation across outcomes related to intrinsic capacity domains were included. Studies involving combination interventions [64,106,107,108] or outcomes not directly related to intrinsic capacity domains were excluded [67,109,110,111,112,113,114,115,116,117,118,119,120,121]. ^#^ IC domain classification was performed by the authors and was not explicitly reported in the original studies. Outcomes were assigned to the locomotor, vitality, sensory, cognitive, or psychological domains according to their conceptual alignment with the WHO Intrinsic Capacity framework [92] and related literature [91,122]. Therefore, the categorization represents a narrative interpretation of the available evidence rather than a direct assessment of intrinsic capacity.

## Data Availability

No new data were created or analyzed in this study. Data sharing is not applicable to this article.
